# A Review of the Bayesian Occupancy Filter

**DOI:** 10.3390/s17020344

**Published:** 2017-02-10

**Authors:** Marcelo Saval-Calvo, Luis Medina-Valdés, José María Castillo-Secilla, Sergio Cuenca-Asensi, Antonio Martínez-Álvarez, Jorge Villagrá

**Affiliations:** 1 University Institute for Computing Research, University of Alicante, 03690 San Vicente del Raspeig, Spain; msaval@dtic.ua.es (M.S.-C.); lmedina@dtic.ua.es (L.M.-V.); sergio@dtic.ua.es (S.C.-A.); amartinez@dtic.ua.es (A.M.-Á.); 2 Centre for Automation and Robotics (UPM-CSIC), 28500 Arganda del Rey, Madrid, Spain; jorge.villagra@csic.es

**Keywords:** ADAS, Bayesian Occupancy Filter (BOF), uncertainty management

## Abstract

Autonomous vehicle systems are currently the object of intense research within scientific and industrial communities; however, many problems remain to be solved. One of the most critical aspects addressed in both autonomous driving and robotics is environment perception, since it consists of the ability to understand the surroundings of the vehicle to estimate risks and make decisions on future movements. In recent years, the Bayesian Occupancy Filter (BOF) method has been developed to evaluate occupancy by tessellation of the environment. A review of the BOF and its variants is presented in this paper. Moreover, we propose a detailed taxonomy where the BOF is decomposed into five progressive layers, from the level closest to the sensor to the highest abstract level of risk assessment. In addition, we present a study of implemented use cases to provide a practical understanding on the main uses of the BOF and its taxonomy.

## 1. Introduction

Nowadays, one of the most important features of Advanced Driver Assistance Systems (ADAS) is the ability to recognize vehicle surroundings. This has led many researchers to propose new ways of evaluating data from sensors in order to reach a high level of understanding relating to pedestrian detection, sign recognition, lane tracking and collision avoidance, among others.

However, the use of commercially available ADAS is generally limited to highways or low speed maneuvers such as parking assistance. Traffic situations in these scenarios are rather simple and the perception system is only required to focus on few, well-defined objects in very structured environments. To broaden their use, such systems need to extend their range of operation to more complex situations in dense urban traffic environments.

To that end, observations from sensor data (that are often noisy) need to be collected on one or more potential obstacles in the vehicle’s environment, to make a robust estimation at each time step of position and velocities. In other words, this is a multi-target tracking problem.

The classical approach is to track different objects independently, by maintaining a list of currently known objects. However the main difficulty of multi-target tracking, known as the data association problem, is that it implies necessary knowledge on (a) whether a new sensor observation corresponds to an existing track or not; (b) whether existing tracks should be maintained or deleted, and whether new tracks should be created. This data association problem is intractable in urban traffic scenarios, which involve numerous appearances, disappearances and occlusions of a large number of rapidly maneuvering targets.

Grid-based representation provides an excellent framework to perform sensor fusion [1]. Different sensor models can be specified to adapt to distinct characteristics of the various sensors, facilitating efficient fusion in the grids. The absence of object-based representation allows easier fusion of low-level descriptive sensory information onto the grids without requiring data association.

Various proposals exist based on environment tesselation, such as the seminal papers of Elfes [2] and Moravec [1], that defined the modeling of sensor readings for the grid, or [3] that uses space sampling for tracking purposes.

Coué et al. [4] presented the Bayesian Occupancy Filter (BOF) to solve the data association problem by evaluating environment occupancy regardless of the nature of the object. A main purpose of the BOF is to permit multiple-sensor data combination, in order to produce a more reliable and robust system in environment recognition. Moreover, the very nature of the method allows data sampling which leads to a more computationally efficient solution. Sensors allowing vehicles to perceive the environment (LIDAR, stereo sensors, sonars, etc.) provide thousands of points in space. Since ADAS require a response within a short period of time, it is necessary to reduce the data dimension while system performance is not endangered. This issue was addressed by a grid-based approach and a probabilistic formulation initially proposed by Elfes in [2], which planted the seeds of the BOF method. One of the most important aspects of the BOF is its intrinsic management of uncertainty via probability and Bayes’ theorem. This allows to handle noise and non accurate sensor data by taking into account their reading model when estimating their cell occupancy. In addition, this model is iteratively refined with history information.

Other approaches, such as [5,6] made use of the evidential framework to update cell occupancy in a grid representation. Indeed, the Dempster-Shafer theory of evidence can be used to filter out inconsistencies between the previous and current grid, considering them as evidences of conflict. The advantage of this approach is the capability of modeling a cell as having been observed neither as free nor occupied. However, finding appropriate values of operators involved is usually a challenging task, which explains why a very reduced number of works follow this technique.

To the best of our knowledge, there is no previous review of the Bayesian Occupancy Filter. Hence in this paper, we review the literature on the BOF and its variants. Moreover, we propose a taxonomy which will help the reader and future researchers understand the levels of applicability of the BOF and its variants reflecting progressive degrees of abstraction, from sensor data to risk prediction.

The paper is structured as follows: we describe the Bayesian Occupancy Filter in Section 2 together with our proposed taxonomy (Section 2.1); in Section 3, we briefly review the specific pre-processing strategies for most relevant sensors used in Advanced Driver Assistance Systems. We present a set of BOF-based refinements, variants and complements found in the literature in Section 4 and Section 5. Following the taxonomy, in Section 6 we describe relevant information on high-level applications. We provide an overall comparison and discussion about BOF variations and use cases in Section 7. Finally, in Section 8 we present our conclusions and some considerations for the future.

## 2. The Bayesian Occupancy Filter

In this section we introduce the Bayesian Occupancy Filter initially proposed by Coué et al. [4,7]. This method was mainly based on two proposals, the Elfes [2] Occupancy Grid idea of tessellating the sensed area, and the Bayesian programming proposal by Coué et al. [8] where they presented a multi-sensor fusion using Bayesian inference.

The concept of the BOF is to create a grid where each cell represents a portion of space. This representation of space is a very generic structure, adapted to both indoor and outdoor environments and to different sensor settings. In its most generic form, cell information can include any relevant information for the application such as occupancy, velocity, danger, reachability, etc. However, information most commonly contained is occupancy and velocity (and also danger information in some applications [9]). For the sake of clarity and consistency with the type of information referred to in state-of-the art papers, occupancy and velocity will hereinafter be the only information stored in each cell.

One of the reasons the BOF was developed was to address the problem of data association, that tends to fail when used with other methods, in complex scenarios where numerous actors/objects are present producing occlusions, appearance and disappearance, and other issues. Moreover, the Bayesian Occupancy Filter (BOF) proposal handles uncertainty and noisy readings by using Bayesian theory [8], which increases the robustness of the system. In addition, the use of the prediction/estimation framework allows to take into account history of occupation and hence overcome occlusion events and substantiate occupancy estimation. However, since there are no notions of objects or behaviors, longer term prediction requires integrating additional prior knowledge such as map information, as addressed in Bayesian Occupancy Filter Using prior Map knowledge (BOFUM).

Another interesting feature is the fact that cells are independent allowing dedicated hardware implementations leading to increased performance. Indeed, the cell independence hypothesis and sensor measurements allow the *for loops* in the Occupancy Grid algorithm to be implemented in a parallel fashion. As a matter of fact, nowadays, with the appearance of modern Multi-Processor System-On-Chips, that incorporate HW accelerators (GPGPUs or FPGAs), real-time implementations of Bayesian Occupancy Filter are quite realistic.

**Figure 1:**
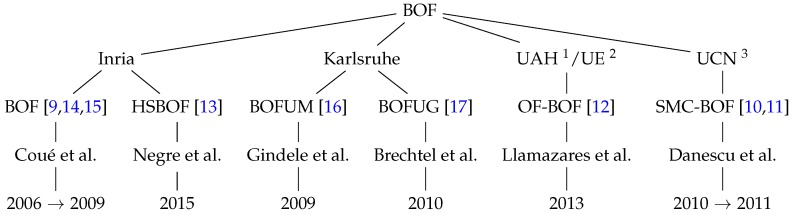
Relationship among Bayesian Occupancy Filter (BOF) techniques, institutions, authors, and publication dates. ${}^{1}$ University of Alcalá de Henares; ${}^{2}$ University of Edinburgh; ${}^{3}$ University of Cluj-Napoca.

Different research groups have faced the problem of perceiving the environment using a Bayesian Occupancy Filter. Figure 1 shows the four main teams that presented works around the BOF, introducing complementary and/or new concepts. INRIA was the first group to develop the Bayesian Occupancy Filter from 2006 to 2009. Since then, other approaches have been carried out by INRIA itself and by different universities and research centers: SMC-BOF [10,11], OF-BOF [12], and HS-BOF [13]. All these variants are analyzed in-depth in Section 5.

### 2.1. BOF-Taxonomy

**Figure 2:**
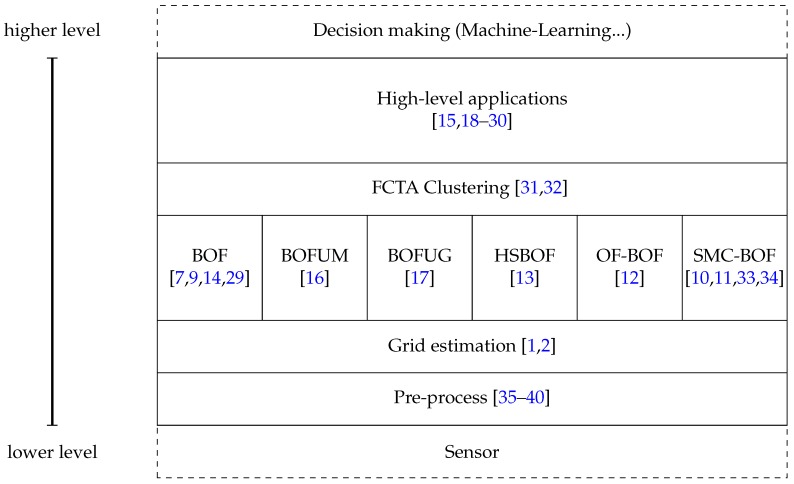
Taxonomy of Bayesian Occupancy Filter.

The Bayesian Occupancy Filter is becoming more and more important in the scientific community. In recent years, the BOF has been intensely studied and it is described in Section 4. First, it is necessary to define a taxonomy to clarify the different layers involved. Figure 2 presents the proposed taxonomy, derived from the review of the state-of–the-art that follows. It involves five main parts, from the first layer at the bottom, which is the closest to the sensor data, to the fifth at the top, where high-level algorithms use grid information to perform complex tasks such as collision predictions. Dashed areas represent previous and posterior steps. Each part of the taxonomy includes articles describing use cases as examples, although others can fit in the taxonomy.

Starting from the bottom layer, the pre-processing involves all data treatment to determine the correspondences between sensor input and cell space. For instance, laser provides obstacle locations in polar coordinates, so previous transformation into Cartesian coordinates is required in order to find the corresponding cell. Moving to the second level, we have the actual BOF and the improvements which have been proposed in the literature. This layer tackles the problem of dynamic objects and egomotion evaluating the occupancy of the cells based on a prediction/estimation paradigm. The next level uses the information of the BOF and its improvements to extract a higher level of information. Concretely, clustering of cells is the union of cells which belong to the same object. This allows a better tracking of the cells to improve prediction and possible occlusions. The highest level of BOF taxonomy relies on all data provided by the lower layers to estimate risks, make decisions, etc. One common application is collision prediction and detection, but others include path optimization, space optimization, etc.

This taxonomy comprises a previous step which is indicated as *Sensor* that refers to all processes in sensor calibration, such as disparity estimation, or the adjustment of data using the intrinsic and extrinsic parameters of the camera. Furthermore, at the top level, the outer part represents high-level methods which abstract from the data to classify behaviors, make decisions, e.g., in the Internet-of-Things concept, this level sends and receives information from the surroundings to create a global network of decisions.

### 2.2. BOF Formal Introduction

In this section, a formal explanation of the Bayesian Occupancy Filter is presented. For further details we refer to [9,29] and the rest of references in this review which provide improvements to the original BOF.

In its early stages of development, the Bayesian Occupancy Filter only took occupation values of the grid into account to determine whether a region is occupied or not. This approach lacks the needed accuracy and, in consequence, an improved version including the velocity of each cell was proposed by [4] under the name of BOF4D. The 4D approach is able to handle overlapped objects with different velocities, although complexity and resources grow exponentially with the size of the grid. This review is centered on BOF4D, the most common in different works (see Figure 1).

Following the description in [23], the 2D Euclidean space is divided in a finite number of cells, each representing a position in the plane. The state of the system O(t) at time *t* is the list of the states of all the cells of the grid: *Occ*, when the cell is occupied or *Emp* if the correspondent space is free. Given a probabilistic sensor model P(z(t)|o(t)) where z(t) is the current observation, the grid is updated following the Bayes’ rule. Under the hypothesis that each cell of the grid is independent from its neighbor cell, each cell state estimation is updated independently [2]. To handle dynamic obstacles, each cell of the BOF maintains not only an estimation of its occupation probability, but also a discretized representation of the probabilistic distribution function (pdf) over velocities. A minimum and maximum velocity value is considered for eventual objects in the space, and the pdf is approximated by a finite histogram over regularly distributed velocity values $v_{n}$ with n∈{1...N}. The discretization step is chosen according to spatial and time discretization: given *q* the size of a cell and *τ* the time step, only integer velocities in terms of $\frac{q}{\tau }$ are taken into consideration. This choice is necessary in order to perform fast and rigorous prediction and updating steps.

The variables used to formalize the BOF probability estimation are as follows:

- *C* is an index that identifies each 2D cell of the grid.
- *A* is an index that identifies each possible antecedent of the cell *c* over all the cells in the 2D grid.
- $Z_{t}\in Z$ where $Z_{t}$ is the random variable of the sensor measurement relative to the cell *c*.
- $v\in V=v_{1},\ldots ,v_{n}$ where *v* is the random variable of the velocities for the cell *c* and its possible values are discretized into *n* cases.
- $O,O^{-1}\in O\equiv occ,emp$ where O represents the random variable of the state of *c* being either *occupied* or *empty*. $O^{-1}$ represents the random variable of the state of an antecedent cell of *c* through the possible motion through *c*. For a given velocity $v_{k}=\left(v_{x},v_{y}\right)$ and a given time step δt, it is possible to define an antecedent for *c* = (*x*, *y*) as $c^{-k}$ = $\left(x-v_{x}\delta t,y-v_{y}\delta t\right)$.

The following expression gives the decomposition of the joint distribution of the relevant variables according to Bayes’ rule and dependency assumptions as state Tay et al. [29]:

$$P\left(C,A,Z,O,O^{-1},V\right)=P\left(A\right)P\left(V|A\right)P\left(C|V,A\right)P\left(O^{-1}|A\right)P\left(O|O^{-1}\right)P\left(Z|O,V,C\right)$$

The parametric form and semantics of each component of the joint decomposition are as follow:

- P(A) is the distribution over all the possible antecedents of the cell *c*. It is chosen to be uniform because the cell is considered reachable from all the antecedents with equal probability. Consequently, given *k* antecedents, each one has a probability P(A) = 1/k.
- P(V|A) is the distribution over all the $\left\{v_{1},\ldots ,v_{n}\right\}$ possible velocities of a certain antecedent of the cell *c*; its parametric form is a histogram.
- P(C|V,A) is a distribution that explains whether *c* is reachable from [*A* = *a*] with the velocity [$V=v\in \{v_{1},\ldots ,v_{n}\}$]. In discrete spaces, this distribution is considered a Dirac with value equal to 1 if and only if $c_{x}=a_{x}+v_{x}\delta t$ and $c_{y}=a_{y}+v_{y}\delta t$, which follows a dynamic model of constant velocity. This Dirac distribution is used in the BOF4D literature, nevertheless, other general distribution approaches could be used.
- $P(O^{-1}|A)$ is the distribution over the occupancy of the antecedents. It gives the probability of the possible previous step of the current cell. Given the antecedents, this probability explains probability that the previous occupancy is reliable with the current antecedents.
- $P(O|O^{-1})$ is the conditional distribution over the occupancy of the current cell, which depends on the occupancy state of the previous cell. It is defined as a transition matrix: $T=\left[\begin{array}{cc} 1-\varepsilon & \varepsilon \\ \varepsilon & 1-\varepsilon \end{array}\right]$, which allows the system to use the null acceleration hypothesis as an approximation; in this matrix, *ε* is a parameter representing the probability that the object in *c* does not follow the null acceleration model.
- P(Z|O,V,C) is the conditional distribution over the sensor measurement values. It depends on the state of the cell, the velocity of the cell and obviously the position of the cell. This models the reliability of a reading in the sensor knowing the current occupancy, and velocities.

**Figure 3:**
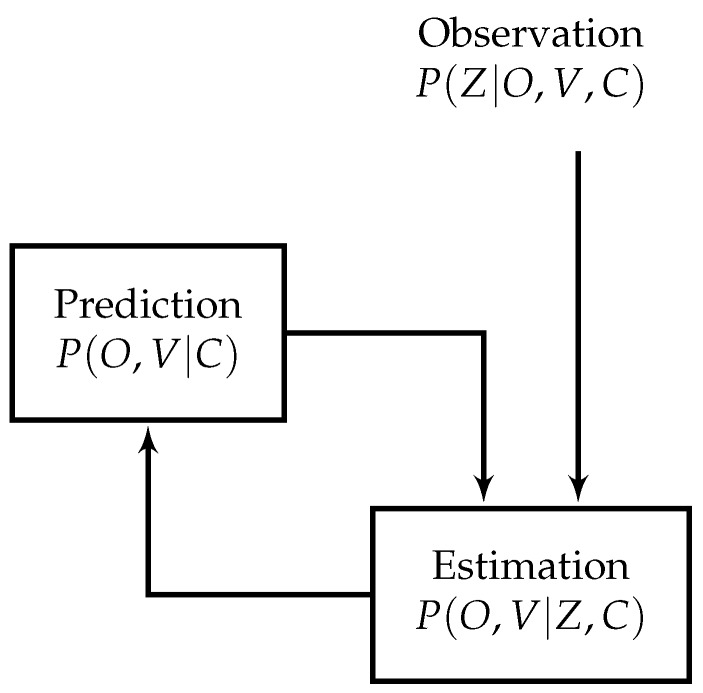
Diagram of prediction and estimation paradigm with the observation input [15]. Reproduced with permission from reference [15]. Copyright 2008 International Journal of Vehicle Autonomous Systems.

The model which calculates the occupancy and velocity is carried out with the following prediction/estimation paradigm depicted in Figure 3.

**Figure 4:**
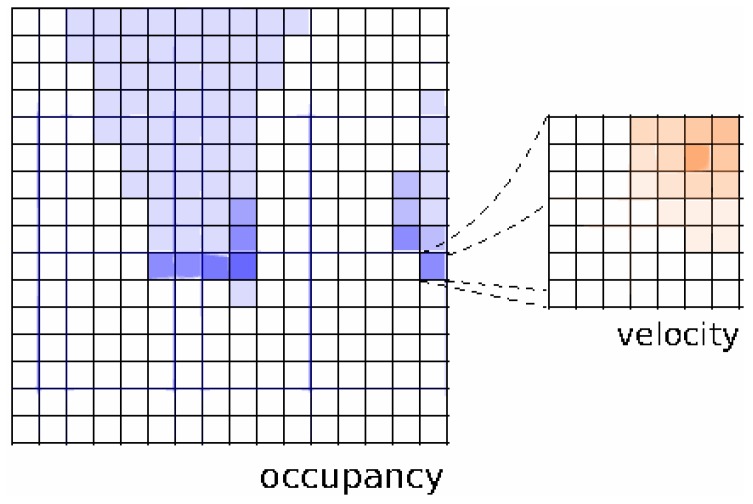
BOF representation from [13]: A two-dimensional grid where each cell has an occupancy value and a histogram of possible velocities. Reproduced with permission from reference [13]. Copyright 2014 IEEE.

The set of possible velocities is discretized. One way of implementing computation of probability distribution is in the form of histograms, as depicted in Figure 4 extracted form [13].

In order to estimate the probability value of occupancy and velocity for one cell, it is possible to decompose joint probability P(O,V|Z,C) into probability of observation and prediction, where the latter takes into account all possible antecedents. These two parts are then calculated and joined to eventually obtain final occupancy and velocity. The whole process is explained in detail in [15,29].

## 3. Sensor Data Pre-Processing

Generally, when we talk about BOF, we assume that information from sensor readings is in the grid space. However, since sensors (Lidar, stereo-vision, ...) do not provide information in this shape, it is necessary to previously treat the data.

Occupancy grids are typically built using range sensors, such as Lidar or ultrasounds. Stereovision has been less frequently used for Occupancy Grid creation, mainly due to its limited accuracy and prohibitive computational effort. However, Perrollaz et al. [36,37,38,39] introduced a novel approach to compute Occupancy Grids from stereo-vision, computing directly in the stereoscopic sensor’s disparity space, using the sensor’s pixel-wise precision during the computation process and allowing the handling of occlusions in the observed area. To that end, it firstly uses the u-disparity approach to avoid processing a large point cloud; then this disparity-space occupancy is transformed into a Cartesian space Occupancy Grid to be used by subsequent applications.

Adarve et al. [35] presented a technique based on Linear Opinion Pool for multi-sensor fusing for Occupancy Grids. The main idea is to have the model of the sensor and term weighting to ensure that non-reliable data does not affect the result. They focused on Lidar and stereo-vision systems, providing all formal aspects which need to be taken into account, such as multi-layer laser reading combination, or partially occluded obstacles.

The approach presented in [4,8] combines multiple sensors using a grid model and based on the Bayesian principle. Further, in [8], modeled sensors work simultaneously, while [4] improves the system taking into account asynchronous sensors.

## 4. Refinements

There are various refinements of the original BOF. In this section, we review the most important variants which include changes in the original BOF to add dynamic information, or computationally efficient approaches. Going further, more significant improvements are presented in Section 5 which approach the BOF from a different point of view, and need to be explained in more detail than the variants.

Regarding the Taxonomy (Figure 2), refinements usually apply to the second layer, although applications can have an effect on higher levels such as collision avoidance in [19]. On one hand, Variants focus on the basics of the method, represented by the BOF box. On the other hand, Improvements and Complements fit in different boxes within the second layer. Nevertheless, the Fast Clustering-Tracking Algorithm (Section 5.5) is an exception since it does not alter the core of the BOF but introduces a higher level of abstraction.

### 4.1. Dynamic Environments

In [14], velocity is expressed as the occupancy difference between time moments, while object detection and tracking is derived from the grid occupancy. With the dynamic model, the term antecedent is introduced to refer to possible occupancy regarding objects which can reach a cell given a certain velocity. In [29], the authors explain the main contributions to the Bayesian Occupancy Filter, by summarizing the original BOF presented by Coué et al. [9], Chen et al. [14], and the posterior formulation in [15] by Tay et al. The main advantage of the Tay paper with regard to Coué’s is the use of velocity information to reduce computational cost, improve predictability and significantly reduce undesired estimation noises.

### 4.2. Collision Cones

In this approach [9], the authors present the original formulation of BOF and apply it to collision avoidance. To do so, they introduce the danger of the cell independently from occupancy. Danger may depend on time to collision and safe traveling distance. An extension of this approach is presented in [24] where the Velocity Obstacle approach is used. The main idea is to create a Collision Cone (CC) which represents the area/velocities where the vehicle will reach the obstacle. In order to take into account obstacle and vehicle velocities, collision danger is calculated by translating the CC using the velocity of the obstacle and evaluating the vector of the vehicle velocity. In the case of multiple obstacles, the union of all Collision Cones is sufficient to calculate the collision free/danger of the vehicle.

Moreover, Coue et al. [9] includes velocity information in two new dimensions ($v_{x}$ and $v_{y}$ for representing the velocities in each direction), which makes the BOF a 4D-BOF. However, Chen et al. [14] include a distribution of velocities in the form of a histogram in the cell, which leads to a 2D-BOF. While the 4D-BOF is capable of representing overlapping objects with different velocities, the 2D-BOF has the advantage of being computationally less demanding and able to infer velocities of cells.

### 4.3. Non-Constant Velocity

Yguel et al. introduces in the report [30] the assumption of non-constant velocity to the BOF framework. This new approach recognizes that cells are regularly updated at frequencies related to their velocity. Therefore, cells with closer velocities are gathered and computed only at certain time steps, while others are ignored. This way, prediction performance is improved in urban scenarios, where object dynamism is usually far from having constant velocity.

**Figure 5:**
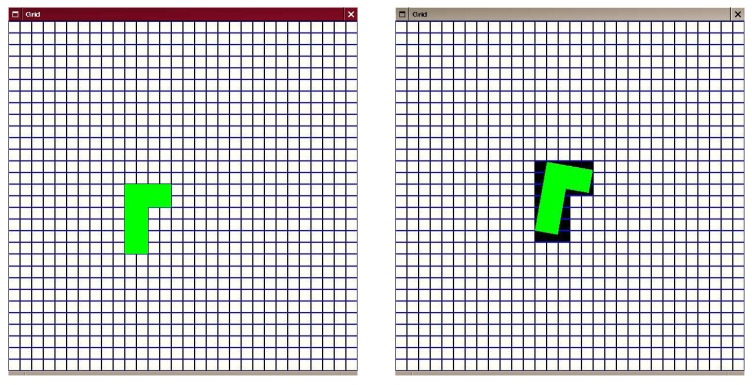
Aliasing problem: the area of an object counted in occupied cell number is not constant for each position of the object in the grid. Reproduced with permission from reference [30]. Copytight 2006 Inria.

Moreover, an object may occupy a different number of cells depending on its orientation, as a cell is occupied regardless of the actual portion of the object lying in the cell space (see Figure 5 extracted from [30]). This aliasing problem is addressed in the aforementioned paper.

### 4.4. Motion Detection

Baig et al. [18,20,21] proposed an improvement of the BOF framework by including a Motion Detection step which evaluates the moving objects in the scene to avoid false positives resulting of egomotion or noisy readings. This Motion Detection is implemented into three steps: Firstly, Free and Occupied Count Arrays keep count of the number of times a cell has been observed as “free” and “occupied”. Secondly, in Counts Update from Previous Step, the egomotion is applied to update the map. Finally, Motion Detection relies on an heuristic applied to estimate which parts are moving.

## 5. Variants and Complements

In this section, we present improvements to BOF consisting in significant additional modifications, for example the use of prior knowledge of the environment to reduce computational time and complexity, or using particle filters to represent the dynamism of cells. Moreover, in this section, we include a clustering proposal which is a relevant proposal despite the fact that it is not a modification aimed at improving BOF performance and features.

### 5.1. BOFUM

**Figure 6:**
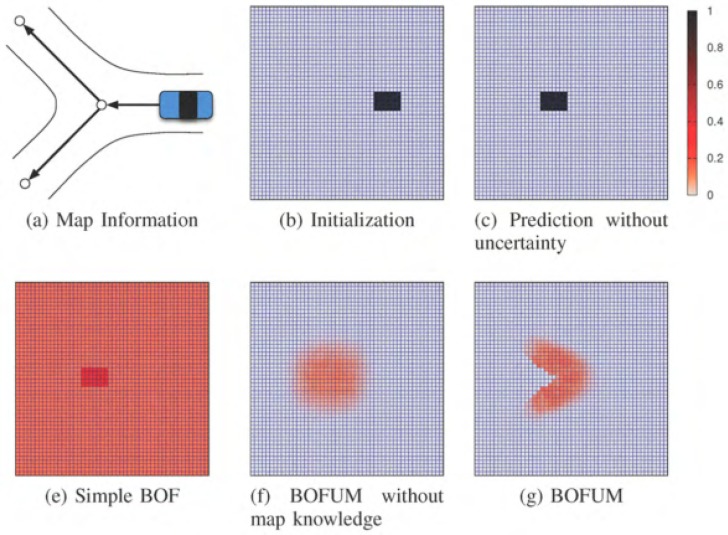
Comparison of prediction results for different filtering techniques after several timesteps proposed by Gindele et al. [16] (Bayesian Occupancy Filter Using prior Map knowledge (BOFUM)). Reproduced with permission from reference [16]. Copyright 2009 IEEE. The image (**a**) shows the knowledge of the environment, (**b**) presents the initial occupancy and (**c**) the prediction without uncertainties. In the second row (**e**) represents the simple BOF prediction using only uncertainties. The BOFUM application is depicted in (**f**) without knowledge and in (**g**) and incorporating the knowledge. The legend shows the occupation probability normalized between 0 and 1.

The algorithm proposed by Gindele et al. [16] is a sophisticated occupancy filter known as Bayesian Occupancy Tracking using prior Map Knowledge (BOFUM). The authors presented a new formulation of the BOF, capable of predicting cell transitions more accurately by enriching the motion model with prior knowledge derived from the cell’s context. They take advantage of the fact that object motion is often heavily dependent on its location in a scene. In the case of traffic scenarios, it is more likely that a car will follow the course of a lane instead of driving perpendicular or on the sidewalks. These behaviour patterns can be anticipated to some degree by looking at the geometric structure of the traffic situation. An example of BOFUM is depicted in Figure 6.

Concretely, BOFUM integrates prior knowledge in the prediction model by using a reachability matrix that shows the probability of an object changing from one cell to another depending on the kind of terrain which represents each cell. In the case of the [16] study, three kinds of terrains were used:

Concretely, the BOFUM integrates the prior knowledge in the prediction model by using a reachability matrix the probability of an object to change from one cell to another depending on the kind of terrain represented by each cell. In the case of Gindele et al. study [16], they use three kind of terrains, being {lane,sidewalk,unknown}. Hence, probability is given by a function and term weights based on three main assumptions:

- Changing the terrain is unlikely due to the robotics or ADAS scenario. The function models the likelihood of one cell being the antecedent of another.
- In the case of moving off the lane: if the target or the antecedent cell are non-lane terrains, it is assumed the object is likely to be a pedestrian, in which case there is no preferred direction and the term weight is set to 1.
- In the case of moving on the lane: vehicles are usually moving along the lane, and the probability of moving out or changing lanes is low, which is modeled using the term weight.

Reachability is defined by an *S* function that models the probability of one cell being an antecedent and a term weight *w* that balances this probability depending on the actual terrain in which we are now.

BOFUM has been used in Brechtel et al. [17], where the authors proposed the use of prior map knowledge to accelerate the convergence of BOF and then potentially reduce required data dimensions. It also features an advanced process model with motion uncertainty, which can be adapted to specialized application needs. In this work, the authors presented an approach for recursively applying importance sampling (IS) to approximate BOFUM calculations. The approach is similar to the well-known particle filter, but from a discrete cell perspective. In their experiments, Brechtel et al. achieved a speedup of at least 40-times by using the IS. Thus, it allows to use the algorithm in real-world applications.

BOFUM was improved by adding movement types in the form of object groups as a property of occupancy in cells. This enables the Bayesian Occupancy Filter using groups (BOFUG) [17] to infer object classes solely from occupancy measurements.

### 5.2. Optical Flow Based Velocity Estimation (OF-BOF)

Llamazares et al. [12] presented a 3D-BOF approach for robot obstacle avoidance, instead of the classical 2D version. It is based on various levels of perception (e.g., three in [12]). Moreover, the authors make use of optical flow techniques to estimate the dynamics of objects, and blob filtering to improve the robustness of perception, noise filtering, and energy consumption efficiency.

Recently, the Bayesian Occupancy Filter is gaining relevance and the number of works using or improving the BOF is growing. This clearly demonstrates the growing interest in this approach for environment perception in the context of driving and robotic applications.

### 5.3. Sequential Monte Carlo Bayesian Occupancy Filter

Danescu et al. [10,11] presented a new approach to Grid Occupancy, making use of Particle filters for calculating the velocity and occupancy of cells. This method, later defined as the Sequential Monte Carlo Bayesian Occupancy Filter (SMC-BOF), is based on three main steps to compute the dynamics of the system: prediction, reallocation of particles depending on the speed and ego-motion of the vehicle; processing measurement, where the sensor data is incorporated into the system to weight the particles and re-sample them; and occupancy and velocity estimation.

Based on SMC-BOF, Nuss et al. [33] proposed a laser and radar data fusion. Later, Oh and Kang [34] modified the SMC-BOF including Linear Opinion Pool to balance the confidence of each sensor with Dempster-Shafer theory to merge the data.

### 5.4. HSBOF

Hybrid Sampling Bayesian Occupancy Filter (HSBOF) is an improvement of the original and the SMC-BOF presented by Negre et al. [13]. This proposal took the fundamentals from the paper [15], including the dynamic model, to handle non-constant velocity and avoid empty cells to move. Compared to the SMC-BOF (Section 5.3), HSBOF does not use particles for static objects while the former estimates the number of particles according to cell occupancy, which is less efficient than Negre’s proposal.

**Figure 7:**
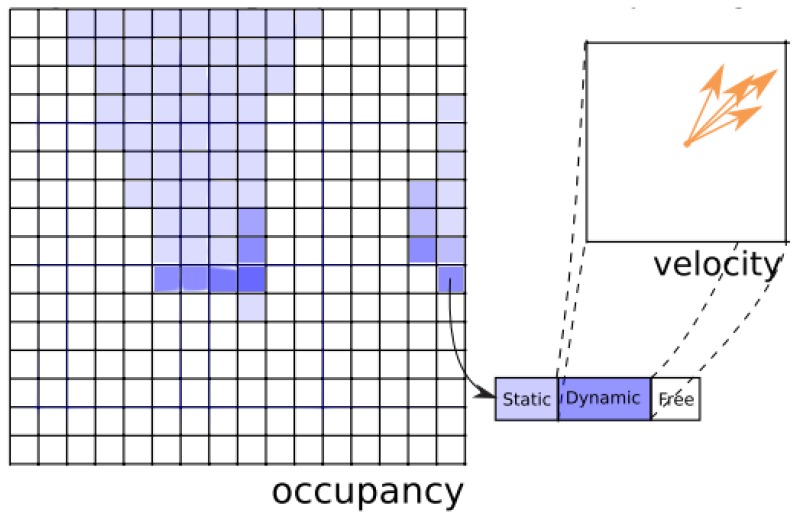
Proposed representation in Hybrid Sampling Bayesian Occupancy Filter (HSBOF) [13]: a two-dimensional grid, to each cell we assigned an occupancy value, a static coefficient P(V=0) and a set of particles drawn along P(V=v|V≠0). Reproduced with permission from reference [13]. Copyright 2014 IEEE.

Negre et al. [13] presented a new representation of the Bayesian Occupancy Filter using a mix of static and dynamic occupancy for describing the environment, as shown in Figure 7. Static occupancy is described with a classic Occupancy Grid [1], while dynamic occupancy has been modeled by a set of moving particles, using the particle filter principle. As the authors stated, “the number of values required to model the velocities have been reduced from a typical 900 per cell to less than 2 per cell in average” and also “the accuracy can be better than in the original BOF as the velocity samples allocation is adaptive and not limited by the grid resolution”.

Rummelhard et al. [28] used HSBOF as the main method to estimate the collision risk prediction for driving applications. Using the dynamics provided by HSBOF, they project the trajectory of the object and evaluate the Time-To-Collision (TTC). This is performed per cell, instead of at object or cluster level to avoid multi-object detection and tracking.

### 5.5. Fast Clustering-Tracking Algorithm

**Figure 8:**
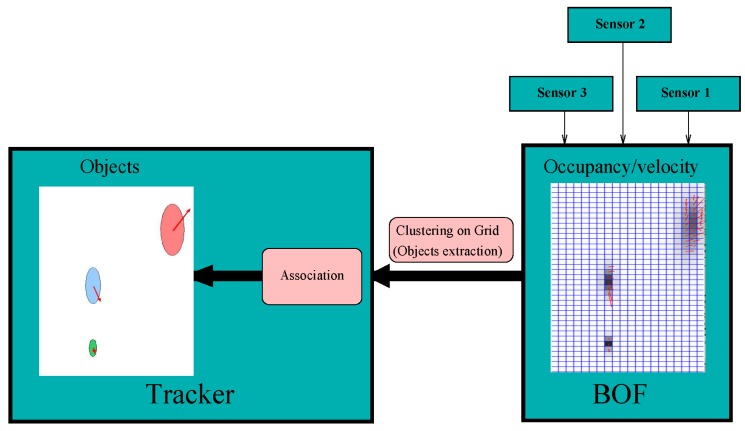
Fast Clustering-Tracking algorithm scheme extracted from Meckhnacha et al. [32]. Reproduced with permission from reference [32]. Copyright 2008 Springer.

This section presents a proposal which does not modify the BOF core but provides a higher-level of abstraction by clustering cells. This proposal, coined as Fast-clustering tracking (FCTA), was presented by Mekhnacha et al. in [25,31,32]. In these papers the authors proposed a fast clustering method extending the idea previously presented in [14]. Figure 8 depicts the FCTA proposal, where the various sensor readings are merged using BOF, and clusterization and tracking are performed to obtain objects. The BOF grid, i.e. the occupancy and the velocity grids, are the input of the object tracker. In order to classify groups or clusters, a classical approach of eight-neighbour cells is used along with occupancy and velocity thresholds to distinguish cells that are certainly occupied. As far as implementation is concerned, the FCTA is performed as a new grid with the same size as the input occupancy one (X,Y), and whose cells have an identifier (ID) for each cluster. To improve performance, the prediction is used to initialize clusters and reduce the search. However, three main situations may occur in the clustering process:

- New object appears, so new identifiers are needed.
- A known cluster.
- Cells with a previous ID are assigned to a new identifier.

The first two situations are normal cases that can be solved in an easy manner with connectivity association. However, the third one is caused by an ambiguous situation due to the closeness of several targets, or by various tracked targets that correspond to one obstacle, which means that a merge is required. In the case of several closed targets, a set of tracks is used to sub-cluster the region. The second ambiguous situation is solved using alias and probability over time to estimate whether the clusters are the same object or not. For further information readers can refer to [25,31].

## 6. High Level Applications and Implementation Aspects

Following the proposed taxonomy, some applications make use of the data provided by BOF to produce useful high level abstraction information. This is the case of pedestrian detection and tracking and collision avoidance. Ros and Mekhnacha [26,27] used the BOF framework to track multiple people from a multi-sensor scenario. In order to determine occupancy, they make use of a generative camera model, based on the idea presented in [22]. They used a hypothesis on the shape of objects to track (these objects were people in this case), and hence, they made a robust system against occlusions using occupancy information on the plane and the images of the cameras.

In [19], Laugier et al. presented a framework for traffic modeling and collision risk assessment. The paper presents the different parts of the sensor, including the formulation to provide a grid map representation from a multi-layer laser sensor and a stereo-vision camera. The framework includes the FCTA (clustering proposal explained in Section 5.5) in order to track objects and thus allow to make a more robust collision evaluation which is developed using Hidden Markov Models and Gaussian Process.

Regarding the implementation of Occupancy Grids in embedded architectures, only a few authors have undertaken the subject. Yguel et al. [41] studied and developed a proposal for multiple data fusion using Graphical Processor Units (GPUs), including polar to Cartesian geometry switch, data fusion, and Occupancy Grid construction. In the course of the experiment, they used Nvidia GeForce FX Go5650 along with a CPU Athlon XP 1900 +. Later, Rakotovao et al. [42,43] presented the implementation of HSBOF on a many-core architecture, concretely an MP-SoC presented in [44], composed of dual core ARM cortex A9 at 800MHz and a many-core accelerator of 64 cores.

## 7. Comparison and Use Cases

Direct comparison of BOF variations is not always an easy task because of their different focuses. However, a set of quality metrics can be identified across reviewed papers as shown in Table 1. This table summarizes advantages and drawbacks according to the following performance indicators:

- Computational cost, considering both the speed and the memory usage.
- Parallelization opportunities, referring to the potential speed-up of the algorithm with HW accelerators.
- Velocity estimation of each cell.
- Robustness against aliasing problems derived of the grid discretization.
- Ability to properly represent the grid empty space.
- Ratio between the accuracy of the cell occupancy probability and the unexpected noise appearing in non-occupied cells.
- Need of prior knowledge of the environment.
- Compactness of the representation model.
- Handling and tracking of moving objects.

All these metrics and their corresponding scores in the table (each "+" symbol means a higher score) provide a qualitative overall view of the strengths of each BOF variant.

Note that the simpler Bayesian Occupancy Filter [9] projects objects on a bi-dimensional grid, based on the assumption that the motion of each individual cell is done at constant speed. This first version of the filter, like most of the variants based on a static grid, allows a good representation of the empty space. This consists in one of its main advantages when compared to object oriented methods that include delicate data segmentation and recognition. Moreover, the Bayesian nature of the BOF enables to intrinsically consider the uncertainties of the world to be represented and of the sensors used, which in addition can be fused quite naturally in a specific spatial representation. Among these representations (Cartesian, polar, column/disparity), the Cartesian grid is the closest one to the real world, and provides very relevant parallelization opportunities in many-core and heterogeneous platforms (GPU, FPGA).

However, this version of the BOF is not able to cope with moving objects because of the absence of an estimation of cell velocity, which results moreover in low occupancy estimation accuracy and significant aliasing problems. To deal with some of these problems, [14,15] proposes a 4D realization of the BOF, where the velocities are either computed as additional states in the filter, or some discrete probabilistic distribution is considered. This family of techniques (named BOF4D in Table 1) keeps or even increases the parallelization opportunities and enhances the handling of moving objects. Nevertheless, the computational cost, both in terms of speed and memory, escalates dramatically.

The BOFUM variant is introduced by [16] with the aim of accommodating speed variation more reliably. To that end, cell transitions are accurately predicted by enriching the motion model with prior knowledge obtained from the grid context. As a result, the number of necessary samples do not depend on the dimension of state space anymore, but on the density of space occupation. This idea allows to significantly improve velocity estimation, and the accuracy of occupancy estimation. The derived representation model is slightly more compact than most classical BOFs, but the price to pay with respect to them is the computational cost, and of course it can only work when a navigation map and a precise localization system are available.

A small variation of BOFUM is presented by the same authors bearing the name of BOFUG [17]. The use of recursive importance sampling, following the scheme of sequential Monte Carlo methods, allows to significantly reduce the computational cost of its predecessor.

Another attempt to compute the cell’s velocity with a restrained cost is the proposal of [12], which uses a camera to apply grid-based optical flow techniques to estimate the motion of each cell. This approach reduces the complexity of BOF4D, but is less prone to be accelerated in heterogeneous platforms, and it does not achieve the same good management of moving objects.

In the SMC-BOF [11], representation of speed probability distribution and estimation of this distribution are no longer a concern. The use of particles instead of cells allows to decouple the assumption that one cell belongs to only one object with only one velocity. In addition, velocity estimation naturally results from the survival or elimination of the particles. The number of particles per cell corresponds to the occupancy of the cell which leads to a very compact model, where the aliasing issues are significantly alleviated, while keeping a very high accuracy/noise ratio. Conversely, free space is represented more poorly with this approach. Another drawback is that despite the fact that the algorithm significantly reduces the computational cost of the filter, it appears to present less parallelization opportunities than its counterparts.

The HSBOF [13] improves speed performance of the SMC-BOF, since a significant number of particles can be discarded to represent static objects. Indeed, the scene is analyzed by fusing a static part with an Occupancy Grid structure, and the dynamic objects, modeled by moving particles. This combination results both in very precise estimation results (in particular with regard to velocity), and in reduced computational cost. Although the data structure is a bit more complex than for SMC-BOF, it is still a more compact model than BOF or BOF4D.

### 7.1. Use Cases

Following the review of the Bayesian Occupancy Filter proposals and their variants, several use cases have been identified. These different uses are reviewed in Table 2. The “X” means the paper (or set of works) has addressed the problem. There are two main parts: the first two columns focus on most common cases in which BOF is applied, which are Autonomous Driving and Robot Guidance. It is worth highlighting that most cases of Autonomous Driving could be applied to robot guidance, since both are moving systems which may interact with dynamic environments.

The last five columns represent other cases: People Tracking, which refers to the use of BOF for evaluating people occupancy of environments; Objects avoidance, which refers to papers that tackle the problem of risk prediction and avoidance; Clustering, which lists articles presenting cell clustering to improve knowledge from the BOF grid; Mapping, which aims at creating an object position map of a full scenario (not only mapping the instant region but having a complete map); Data fusion, corresponding to works that facilitate data fusion using BOF. Concerning these latter cases, Data Fusion is, along with Object Avoidance, one of the most used, since it was originally one of the main reasons to develop the grid-based approach. Nevertheless, some works have addressed People Tracking, which represents a completely different usage. Mapping has been studied, but only in a few works. Clustering, has also been studied to provide a higher level of understanding to the tessellated environment.

## 8. Concluding Remarks

In this paper, we presented a review of the Bayesian Occupancy Filter (BOF) method together with its variants and improvements. BOF describes the environment of a vehicle or robot in a tessellated grid in order to provide occupancy and velocity information of objects presented in this area. Bayesian rules are used to estimate and predict the dynamics, including egomotion, in order to robustly detect occupied regions for collision avoidance or risk detection, among other possible purposes. We proposed a taxonomy of BOF to give a clear picture the different levels of abstraction and possible approaches at each level, which is an important contribution for readers and future researchers, because it can help them clearly determine the different parts of the process. Moreover, we presented the main research groups that have used BOF or have proposed variants and improvements.

In the review, we distinguished different proposals and divided them into refinements or variant/complements. Refinements refer to works where the BOF is modified with the addition of new features that make it more robust or that allow taking into account different aspects. Variant/complements refer to significant changes where the core of the BOF is different (e.g., particle filter instead of histogram of velocities, reachability information in cells, etc.). In addition, we presented other relevant works which made use of BOF, or proposed embedded systems to improve performance. These variants were discussed and compared on the basis of a set of relevant metrics providing the reader with an overall quality view of each approach.

Finally, we analyzed the use cases and showed that most proposals focus on autonomous driving followed by robot guidance. Based on this review, we conclude that BOF has been mainly applied in data fusion, clustering and object avoidance. Nevertheless, because of their generality, BOF proposals allow further improvements related to adaptation to different situations and new use cases. For instance, tessellation with different cell sizes might improve performance, and crowd analysis could be an interesting new application scenario. 

## Figures and Tables

**Table 1:** Key performance indicators.

	BOF	BOF4D	BOFUM	BOFUG	OF-BOF	SMC-BOF	HSBOF
Computational cost	++	0	+	++	+	++	+++
Parallelization opportunities	++	+++	++	++	+	+	++
Velocity estimation	0	++	+++	+++	++	+++	+++
Aliasing/discretization robustness	0	0	++	++	0	+++	+++
Empty space representation	+++	+++	+++	+++	+++	+	+++
Accuracy/noise ratio	+	+	++	++	+	+++	+++
Prior knowledge needed	+++	+++	0	0	+++	+++	+++
Representation model compactness	+	+	++	++	+	+++	++
Moving objects handling	0	++	+++	+++	+	+++	+++

**Table 2:** Use cases of Bayesian Occupancy Filter.

	Auto.	Robot	People	Object	Clustering	Mapping	Data
	Driving	Guidance	Tracking	Avoidance			Fusion
Adarve et al. [35]	X						X
Baig et al. [18,20,21]	X				X		
Brechtel et al. [17]	X				X		
Chen et al. [14]			X		X		
Coue et al. [4,7,8]	X						X
Coué et al. [9]	X			X			
Danescu et al. [10]	X			X			
Danescu et al. [11]	X			X	X		
Elfes [2]		X				X	X
Fleuret et al. [22]			X				X
Fulgenzi [23]	X			X	X	X	X
Fulgenzi et al. [24]	X			X			
Gindele et al. [16]	X						
Laugier et al. [19]	X			X			X
Llamazares et al. [12]		X		X			
Mekhnacha et al. [31,32]	X				X		
Mekhnacha and Raulo [25]	X			X	X		
Moravec [1]		X				X	X
Negre et al. [13]	X						
Nuss et al. [33]	X						X
Oh and Kang [34]	X						X
Perrollaz et al. [36,37,38,39]	X						
Ros and Mekhnacha [27]			X	X	X		
Ros and Mekhnacha [26]			X	X			
Rummelhard et al. [28]	X			X			
Tay et al. [15]			X		X		
Tay et al. [29]	X		X	X			
Yguel et al. [30]				X			
Yoder et al. [40]	X						X
